# A distinct and reproducible teleconnection pattern over North America during extreme El Niño events

**DOI:** 10.1038/s41598-024-52580-9

**Published:** 2024-01-30

**Authors:** Margot Beniche, Jérôme Vialard, Matthieu Lengaigne, Aurore Voldoire, Gangiredla Srinivas, Nicholas M. J. Hall

**Affiliations:** 1 LEGOS, CNRS/CNES/IRD/Université de Toulouse, Toulouse, France; 2 LOCEAN-IPSL, CNRS/IRD/MNHN/Sorbonne Université, Paris, France; 3 MARBEC, CNRS/IFREMER/IRD/Université de Montpellier, Sète, France; 4 CNRM, Météo-France/CNRS/Université de Toulouse, Toulouse, France; 5 CSIR-National Institute of Oceanography, Dona Paula, Goa India

**Keywords:** Atmospheric science, Atmospheric dynamics

## Abstract

El Niño-Southern Oscillation (ENSO) teleconnections are an important predictability source for extratropical seasonal climate forecasts. Previous studies suggest that the ENSO teleconnection pattern depends on the ENSO phase (El Niño vs. La Niña) and/or Sea Surface Temperature (SST) pattern (central Pacific vs. eastern Pacific El Niño events). Observations and ensemble simulations with the CNRM-CM6.1 atmospheric general circulation model indicate that only extreme El Niño events (e.g. 1982–1983, 1997–1998, 2015–2016) display a statistically significant eastward shift relative to the well-known Pacific-North American teleconnection pattern that occurs during both central and eastern Pacific moderate El Niño or during La Niña. This specific teleconnection pattern emerges when equatorial SST anomalies are both eastward-shifted and sufficiently large to exceed the deep atmospheric convection threshold over most of the eastern Pacific, resulting in a basin-wide reorganization of tropospheric heat sources. It yields> 0.5 std wet conditions over Western United States (74% likelihood) as well as> 0.5 std warm anomalies over Canada and the Northern United States (71% likelihood), with more consistency across events and ensemble members than for any other El Niño or La Niña type. These findings hold important implications for the seasonal forecasting of El Niño’s impacts on the North American climate.

## Introduction

El Niño-Southern Oscillation (ENSO) is the dominant driver of year-to-year climate variability^1^, with irregular warm (El Niño) and cool (La Niña) Sea Surface Temperatures (SST) anomalies occurring every 2 to 7 years in the equatorial central and/or eastern Pacific. These anomalies generate rainfall and tropospheric latent heat release, leading to the formation of Rossby waves^2^ that induce seasonal precipitation, wind and surface temperature anomalies known as teleconnections along their path to the extratropics^3,4^. As a result, ENSO affects the climate on a global scale, causing cold spells and heat waves, floods and droughts, with the attendant societal consequences^5^. ENSO teleconnections are a primary source of predictability for extratropical seasonal climate due to the improved accuracy of ENSO forecasts from dynamical models^6^.

**Figure 1:**
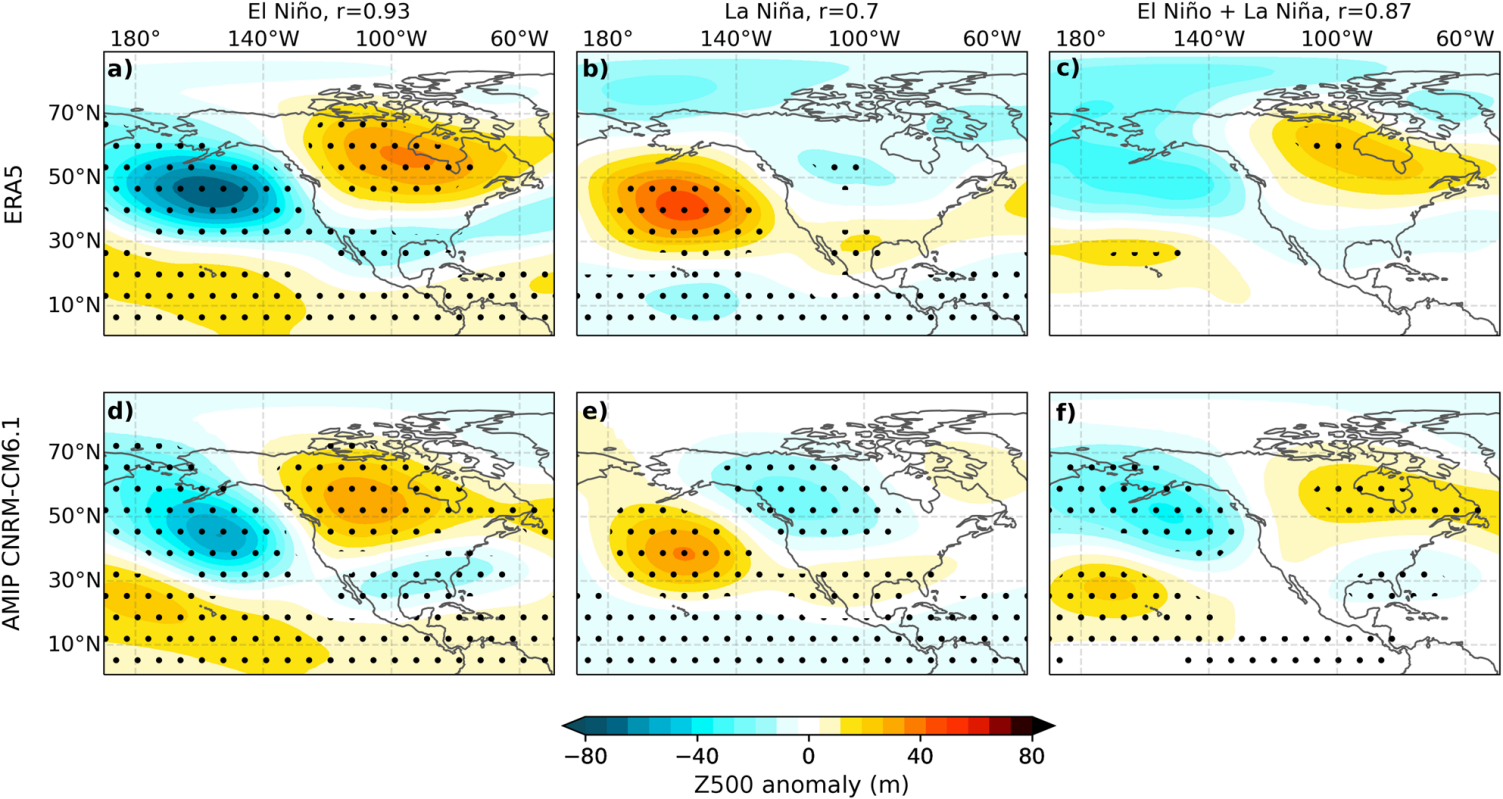
Composites of December–February (DJF) 500 hPa height anomaly (shaded, m) over the 1979-2019 period for El Niño (**a**,**d**), La Niña, (**b**,**e**) and El Niño+La Niña (**c**,**f**). The top row displays composites based on ERA5 data, while the bottom row illustrates composites from the CNRM-CM6.1 AMIP-type historical 6-member (CTL) ensemble mean. The composites are not normalized. r is the correlation coefficient between the observed and corresponding modelled composite. Dots indicate regions where anomalies exceed the 97.5% confidence level based on a bootstrap test (1000 resampling for observations, 10,000 for the model).

The Pacific-North America (PNA) pattern^7^ is widely recognized as the dominant ENSO teleconnection pattern^8^. During El Niño events, a positive PNA pattern is usually observed. Its distinctive features include below-normal geopotential height south of the Aleutian Islands and the southeastern United States (US), while above-normal heights are observed over the US-Canadian border^9^ (Fig. 1a). This pattern provides abnormally warm winter temperatures to Northwestern North America and unusually wet, cool conditions in the southern US^10^. El Niño and La Niña exhibit notable asymmetries in their tropical Pacific oceanic characteristics and dynamics^11^ and in their atmospheric teleconnections^12,13^. Observed El Niño mid-level geopotential height composites anomalies (Fig. 1a) display stronger signals compared to those of La Niña and an above-normal geopotential height shifted eastward over Northern America by approximately 15^∘^ (Fig. 1b).

As a result, surface temperature and rainfall signals are not symmetrical (Fig. 1c) over North America between the two ENSO phases^14,15^. This teleconnection asymmetry has been attributed to nonlinearities in the response of tropical convective precipitation to SST anomalies^12,16,17^, as SSTs above 27^∘^C-28^∘^C are a critical prerequisite for initiating tropical convection^18,19^. In the equatorial Pacific, the climatological SST increases towards the west, crossing this tropical convection threshold near the dateline. Positive anomalies in this area lead to increased rainfall east of the dateline, while negative anomalies suppress rainfall west of the dateline. This mechanism accounts for the larger and eastward-shifted rainfall anomalies and associated atmospheric response observed during El Niño^12^.

The ENSO SST pattern itself also introduces a source of asymmetry in the teleconnection pattern. Notably, El Niño displays stronger SST anomalies that are shifted eastward relative to La Niña^11,20^. In addition to this El Niño-La Niña asymmetry, the maximum SST anomaly location also varies between events, characterized by Central Pacific (CP, also known as “Modoki”, “Warm Pool” or “dateline”) and Eastern Pacific (EP, “Cold Tongue” or “Conventional”) events^21^. This inter-event variability is commonly referred to as ENSO diversity^22–26^ and is more pronounced for El Niño than for La Niña. EP and CP events have been suggested to trigger different teleconnection patterns^27–31^. This leads to fundamental questions: Are El Niño and La Niña teleconnection patterns fundamentally different, or do most of the disparities between them stem from differences between CP and EP teleconnection patterns? Additionally, what are the SST pattern diversity and atmospheric nonlinearities relative contributions in the change of teleconnection patterns? To the best of our knowledge, only one study^32^ has investigated this last matter, concluding that SST pattern diversity and atmospheric nonlinearities both contribute to teleconnection patterns asymmetries.

A relationship exists between ENSO diversity and its amplitude, wherein extreme El Niño events are consistently EP events. However, it is important to highlight that not all EP events exhibit large amplitudes, there are also instances of moderate EP El Niño events^33^ (e.g. 1986–1987, 1991–1992 or 2006–2007). Some studies have emphasized the amplitude of El Niño, rather than its diversity, as a primary driver of teleconnection diversity, and specifically that only extreme El Niño events (e.g., 1982–1983, 1997–1998 and 2015–2016, see “Methods” section for the definition of extreme El Niño events) increase the likelihood of wet conditions in California^34,35^. To achieve this effect, SST anomalies must reach a sufficient magnitude to promote deep atmospheric convection in the eastern Pacific, thus reshaping the ENSO teleconnection pattern^36–38^. Based on observational data, Chiodi and Harrison^36,37^ argued that El Niño events accompanied by convective perturbations in the eastern-central Pacific (160^∘^W to 110^∘^W) yield more statistically-significant teleconnections than other El Niño events. Johnson and Kosaka^38^ refined these results using ensemble Atmospheric General Circulation Model (AGCM) experiments, identifying statistically significant differences in teleconnection patterns between El Niño events with and without convective perturbations in the eastern-central Pacific.

Previous studies have thus attributed the observed diversity in the ENSO teleconnection pattern to various factors, including El Niño-La Niña asymmetries^12,16^, differences between the CP and EP El Niño SST patterns^27–29^, the presence of convection in the eastern-central Pacific^36–38^and, concerning Californian rainfall, the occurrence of an extreme El Niño^34,35^. However, attributing ENSO teleconnection diversity to a specific event type in observations is challenging due to the considerable uncertainties in the teleconnection pattern itself, stemming from the limited number of events and large mid-latitude internal atmospheric variability^39^. This underscores the importance of complementing observational analyses with AGCM ensemble experiments, enabling a clear separation between the forced response and atmospheric noise^39–41^. The signal-to-noise ratio can vary depending on the location and amplitude of the teleconnection pattern. A more reproducible teleconnection pattern, characterized by a higher signal-to-noise ratio, implies greater predictability potential. To our knowledge, no studies have investigated the reproducibility of the various ENSO (La Niña, El Niño, CP, EP, etc...) teleconnection patterns. This objective will also be a focus of this study.

This article uses a combination of observations and ensemble experiments performed with the atmospheric component of the CNRM-CM6.1 climate model (Srinivas et al., submitted^42^) to investigate the factors contributing to the diversity of northern hemisphere ENSO teleconnections during boreal winter. The first section of our results highlights the distinctive nature of the teleconnection pattern associated with extreme El Niño events. Subsequently, we unravel how SST pattern diversity and atmospheric nonlinearities interact to produce this unique teleconnection pattern. In the last results section, we demonstrate that the extreme El Niño teleconnection pattern is more reproducible across events and ensemble members than any other type of ENSO event. In the “Discussion” section, we compare our results with previous studies, and discuss their implications for seasonal forecasting.

## Results

### Uniqueness of the extreme El Niño teleconnection pattern

We initiate our analysis by evaluating the ability of the CNRM-CM6.1 atmospheric component to replicate the observed El Niño and La Niña teleconnection patterns, including asymmetries outlined in the introduction (Fig. 1a–c). The composite geopotential height anomaly pattern for the model (Fig. 1d,e) is computed over the same time frame and for the same events as observations, using six ensemble members. The model reproduces the patterns and amplitudes of the observed La Niña and El Niño teleconnections reasonably well, achieving pattern correlations of 0.7 and 0.93, respectively. Notably, the model successfully captures the higher amplitude of El Niño teleconnections and replicates the eastward shift of the pattern over North America. Overall, the atmospheric component of the CNRM-CM6.1 model reasonably reproduces the El Niño-La Niña teleconnection asymmetries (Fig. 1c,f).

**Figure 2:**
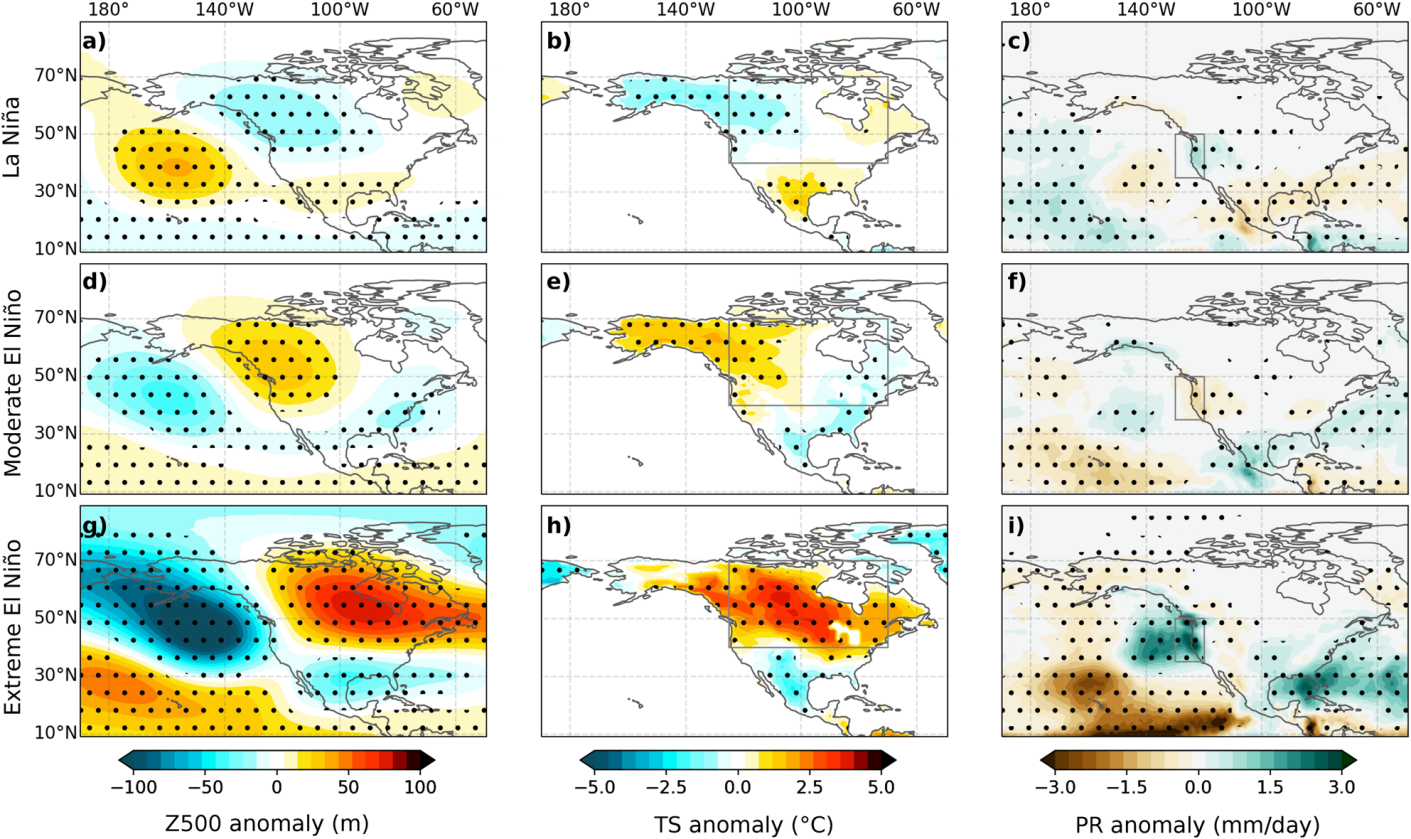
Composites of (left column) 500 hPa height (shaded, m), (middle column) land surface temperature (shaded, ^∘^C) and (right column) rainfall (shaded, mm/day) December-February (DJF) anomalies from the ensemble-mean CTL experiment over the 1979–2019 period for (top row) La Niña, (middle row) moderate El Niño and (bottom row) Extreme El Niño events. The composites are not normalized. The North American box (40–70^∘^N, 125–80^∘^W) used in Fig. 6c is marked in the middle column, and the Western United States box (35–50^∘^N, 130–120^∘^W) used in Fig. 6b in the right column. Dots indicate regions where anomalies exceed the 97.5% confidence level based on a bootstrap test with 10,000 resamples.

**Figure 3:**
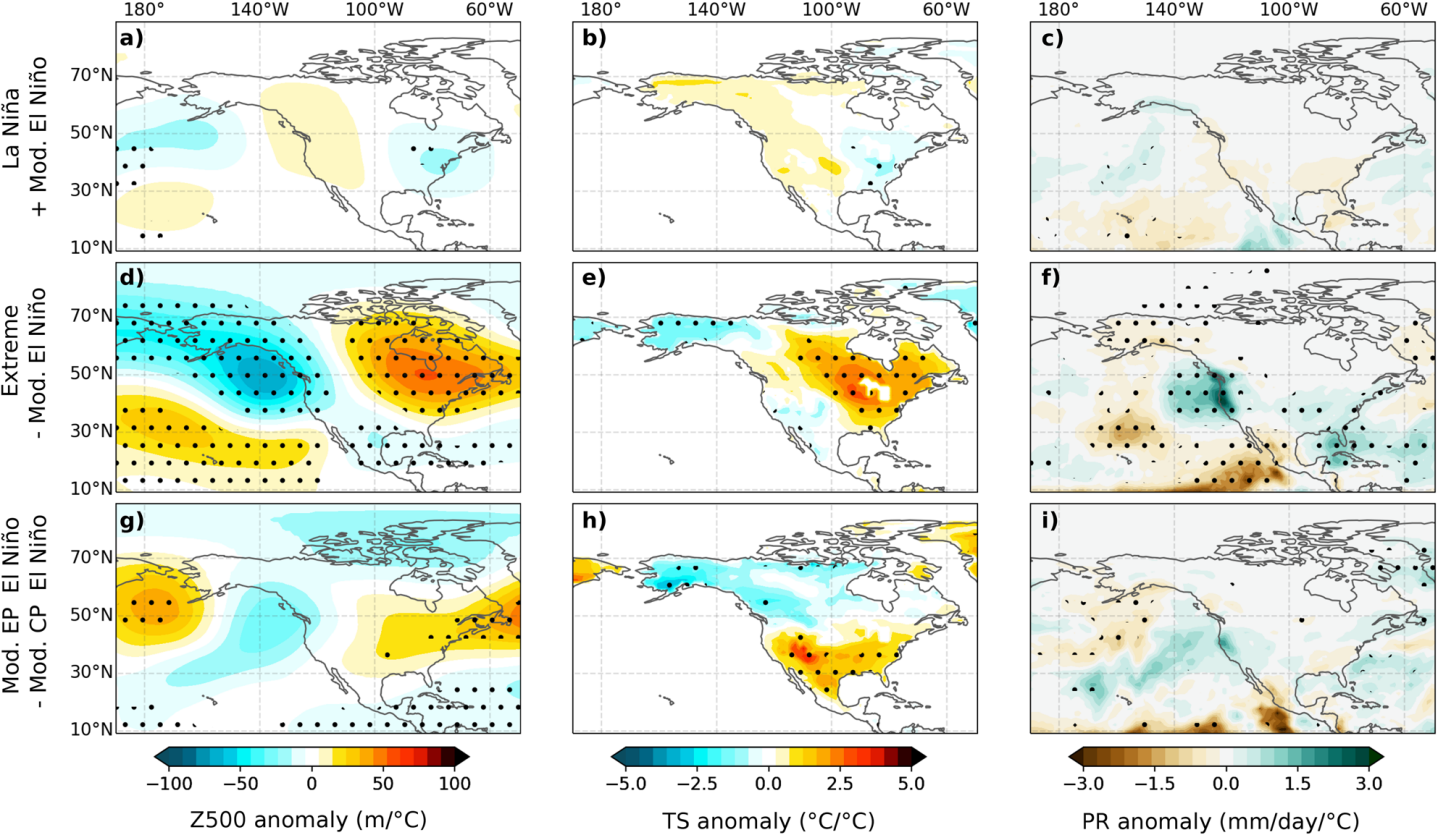
Composites sum/differences of (left column) 500 hPa height (shaded, m/^∘^C), (middle column) land surface temperature (shaded, ^∘^C/^∘^C) and (right column) rainfall (shaded, mm/day/^∘^C) December–February (DJF) anomalies from the ensemble mean CTL experiment over the 1979–2019 period for (top row) La Niña plus El Niño, (middle row) Extreme minus moderate El Niño and (bottom) moderate Eastern Pacific (EP) minus moderate Central Pacific (CP) events. Each composite is obtained after normalizing by SST anomalies in the forcing region ([5^∘^N/S, 170^∘^E-130^∘^W], see “Methods” section) in order to emphasize pattern rather than amplitude differences. Dots indicate areas where anomalies exceed the 97.5% confidence level based on a bootstrap test with 10,000 resamples.

We now explore the factors contributing to the diversity of the teleconnection pattern. Figure 2 displays model composites of 500 hPa geopotential height (Z500), land surface temperature (Ts) and rainfall (Pr) anomalies for La Niña, moderate El Niño and extreme El Niño events (refer to the “Methods” for event category definitions) during December-February (DJF). Moderate El Niño composites (Fig. 2d–f) exhibit the canonical positive PNA pattern, featuring a strengthened Aleutian low, elevated pressure over western Canada, increased rainfall over the subtropical North Pacific (160^∘^W; 35^∘^N), decreased rainfall on the west American coast, warm anomalies over Alaska and western Canada and cold anomalies along the Gulf of Mexico coastline. The La Niña composite (Fig. 2a–c) closely mirrors the opposite of the moderate El Niño composite (Fig. 2d–f), both in terms of amplitude and pattern. To focus on pattern changes rather than amplitude changes, Fig. 3a–c depicts the sum of normalized composites for moderate El Niño and La Niña (see methods for details and justification of the normalization). It convincingly demonstrates that there are no statistically significant disparities between the teleconnection patterns associated with moderate El Niño and La Niña events, whether examining Z500, Ts or Pr. These findings are corroborated with observations for Z500 and Ts from ERA5 and Pr from GPCPv2.3 (Fig. S2). The striking similarity between the mirror-image teleconnection patterns for moderate El Niño and La Niña strongly suggests that El Niño-La Niña teleconnection asymmetries over North America, as depicted in Fig. 1, are primarily driven by extreme El Niño events.

Additional evidence in Fig. 2 underscores the distinctiveness of teleconnection patterns associated with extreme and moderate El Niño. Notably, the extreme El Niño patterns exhibit higher amplitude than the moderate El Niño composite across all variables, and a noticeable eastward shift of the entire teleconnection patterns. Specifically, the north Pacific negative Z500 anomaly is shifted approximately 20^∘^ eastwards, with negative anomalies touching the North American coast, while the positive Z500 anomaly over North America displays a more pronounced eastward shift of about 30^∘^, extending all the way to the western North Atlantic (Fig. 2g). Accordingly, the surface warming over North America shifts southeastward, with the warmest anomalies over central and eastern Canada and the northern United States, as opposed to Alaska. The positive precipitation patch over the subtropical Pacific, typical during moderate events, also shifts eastwards during extreme El Niño, impacting the western US coast, including California, consistent with previous findings^34,35^. Although noisier, the observed extreme El Niño teleconnection patterns from ERA5 (Fig. S2g-i) aligns with our simulation results. Additionally, Fig. 3d presents the composite teleconnection difference between extreme and moderate El Niño, with the normalization allowing a focus on the pattern rather than the amplitude. While no statistically significant asymmetries were found between moderate El Niño and La Niña (Fig. 3a–c), there are statistically significant differences between extreme and moderate El Niño for all variables (Fig. 3d-i), including the eastward shift of the Z500 signal, the warming of eastern North America, and the eastward shift of the rainfall towards the US west coast.

The ENSO teleconnection pattern displays notable differences solely for extreme El Niño events, i.e. the three strongest Eastern Pacific (EP) events since 1982. This suggests that the different teleconnection patterns between CP and EP events discussed in earlier research^27–31^ primarily stems from extreme events, and that there are no differences between moderate EP and CP events teleconnections. To investigate this aspect, Fig. 3g–i illustrates the differences in teleconnection patterns between moderate EP and CP events. Although some statistically significant differences emerge (e.g., Z500 over the North Pacific, over the western North Atlantic and Ts over Mexico; see “Discussion” section), there is no statistically significant eastward shift for moderate EP events teleconnections over North America, as is evident for extreme El Ni.o events. This confirms that the change in teleconnection pattern, as identified in the aforementioned studies, are predominantly associated with extreme El Niño events. In the “Discussion” section, we will emphasize that our classification of “ EP events” closely aligns with what some studies^36–38^ have defined as “convective eastern Pacific” events, i.e. that only the strongest convective perturbations corresponding to extreme El Niño events induce a shift in the teleconnection pattern.

This section demonstrated that both La Niña and moderate El Niño, whether of CP or EP types, consistently exhibit the well-established PNA teleconnection pattern in observations and the CNRM-CM6.1 atmospheric component. Our findings point to the occurrence of an extreme event as the key driver of a change in the ENSO teleconnection pattern, rather than the ENSO phase, diversity or the occurrence of convection in the eastern-central Pacific. This peculiar pattern resembles the negative phase of the Tropical/Northern Hemisphere (TNH) pattern^43,44^.

### Eastward-shifted SST forcing and atmospheric nonlinearities joint contributions to the extreme El Niño teleconnection pattern

**Figure 4:**
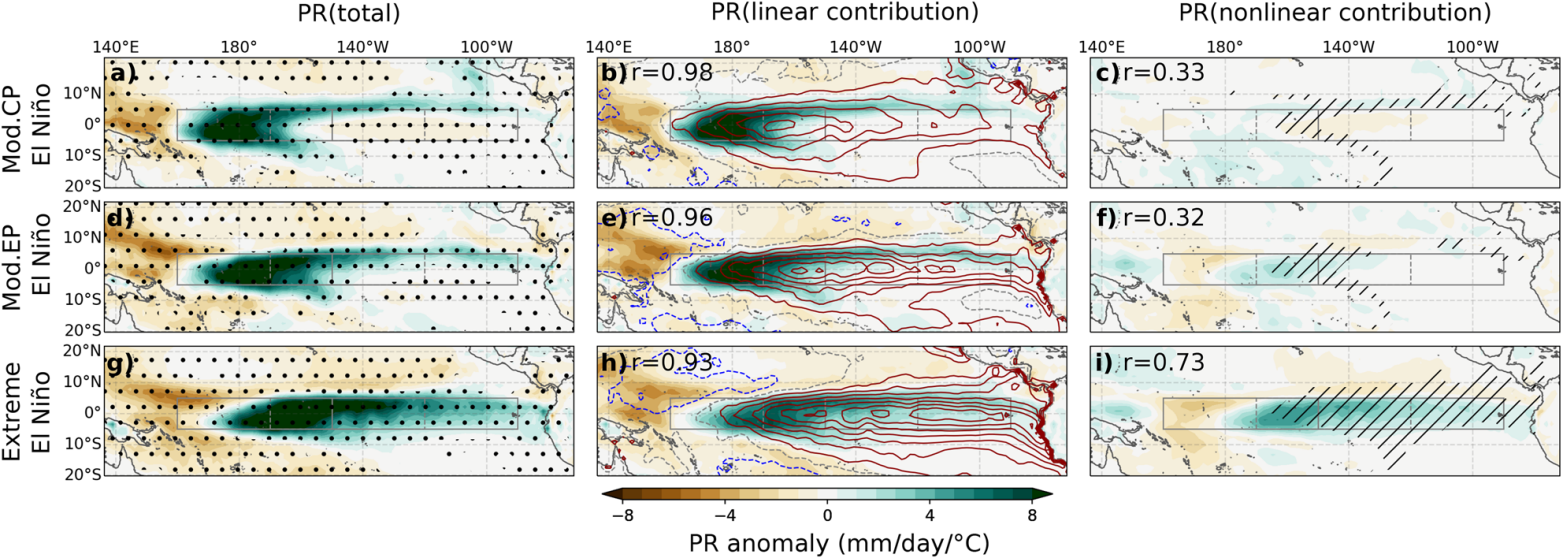
Composites of December–February (DJF) tropical Pacific rainfall anomalies (shading, mm/day/^∘^C) from the ensemble mean CTL experiment over the 1979–2019 period for: (top row) moderate Central Pacific (CP), (middle row) moderate Eastern Pacific (EP) and (bottom row) Extreme El Niño events. Composites (shading, mm/day/^∘^C) have been obtained after normalizing fields by the average [5^∘^N/S, 170^∘^E–130^∘^W] SST anomalies (considered the main forcing region, see “Methods” section), in order to highlight the pattern rather than the amplitude differences. The left column displays the total anomaly from our 6-member ensemble simulation, while the middle and right column respectively display the linear and nonlinear response to SST contributions (see “Methods” section for details). Dots in the left column indicate areas where anomalies exceed the 97.5% confidence level based on a bootstrap test with 10,000 resamples. On middle panels, contours (red for positive, blue for negative, with a 0.2 ^∘^C contour interval) indicate the corresponding SST composites. Hatching in the right column indicates when the SST anomaly brings total SST from below to above the deep convection threshold (27.5^∘^C). On the middle (right) column, r indicates the pattern correlation between the total anomaly and the linear (nonlinear) SST contribution to the rainfall anomaly. Black continuous boxes encompass the Niño3 and Niño4 regions. The black dashed box highlights the Niño3.4 region.

In this section, we investigate the relative roles of the SST pattern and atmospheric nonlinearities in establishing the specific extreme El Niño teleconnection pattern. As discussed in the introduction, the nonlinear part of the atmospheric response can induce teleconnection asymmetries in response to a symmetrical SST forcing. But the linear atmospheric response to the ENSO asymmetrical SST forcing will also induce teleconnection asymmetries. We will thus estimate the relative roles of the linear and nonlinear atmospheric response to SST in establishing teleconnection asymmetries, and more specifically the extreme El Niño teleconnection pattern. We adopt the approach outlined by Srinivas et al. (in revision)^42^. It involves conducting two 6-member ensemble experiments: one follows the standard AMIP-style ensemble experiment, where the atmospheric response is driven by observed SST anomalies, while the other experiment involves reversing the sign of SST anomalies. By doing so, it allows diagnosing the linear and nonlinear parts of the atmospheric response to SST (see “Methods” section for details). Despite different localization of the equatorial SST anomalies, CP and moderate EP events exhibit similar teleconnection patterns, while only extreme EP events display a distinct teleconnection pattern. To understand the reasons underlying this feature, Fig. 4 displays the SST patterns (contours on middle column), tropical rainfall response (left column) and its linear and nonlinear contributions (middle and right columns, respectively) for CP, moderate EP and extreme El Niño events.

The SST pattern associated with moderate CP events (Fig. 4b, red contours) features warm anomalies over the Niño3.4 and Niño4 regions, with maximum rainfall anomalies occurring over Niño4 (Fig. 4a). During moderate EP events, the SST anomalies are slightly shifted eastwards, with a peak in Niño3.4, and become stronger in the eastern Pacific, as expected (Fig. 4e, red contours). Despite this distinct SST pattern, the maximum rainfall anomalies location during moderate EP event does not greatly differ from that of moderate CP events (Fig. 4a,d). Figure 4b,e indicates a relatively linear rainfall response during moderate CP and EP events, in agreement with Srinivas et al. (in revision)^42^. The larger eastern Pacific SST anomalies during moderate EP events (contours in Fig. 4e) are indeed insufficient to trigger deep convection (hatching on Fig. 4c,f) over the climatology cool Niño3 region, thus resulting in a relatively small nonlinear contribution to rainfall there (shading in Fig. 4f). Conversely, extreme El Niño events are characterized by a much larger warming over Niño3 (contours on Fig. 4h). During these events, the maximum rainfall response shifts eastward to  160^∘^W, and positive rainfall anomalies extend along the equator all the way to the South American coast (Fig. 4g). The linear and nonlinear components contribute roughly equally to rainfall anomalies over Niño3. This can be attributed to the SST threshold required to initiate deep atmospheric convection. The Niño3 SST anomalies are much larger for extreme than for moderate El Niño events (contours in Fig. 4e,h), now exceeding the convective threshold and yielding a large nonlinear rainfall response over Niño3 (hatching and shading on Fig. 4i, respectively). In summary, Fig. 4 demonstrates that only extreme El Niño events exhibit sufficiently large SST anomalies over Niño3 to exceed the convective threshold and induce a large eastward shift of the tropospheric heat source.

**Figure 5:**
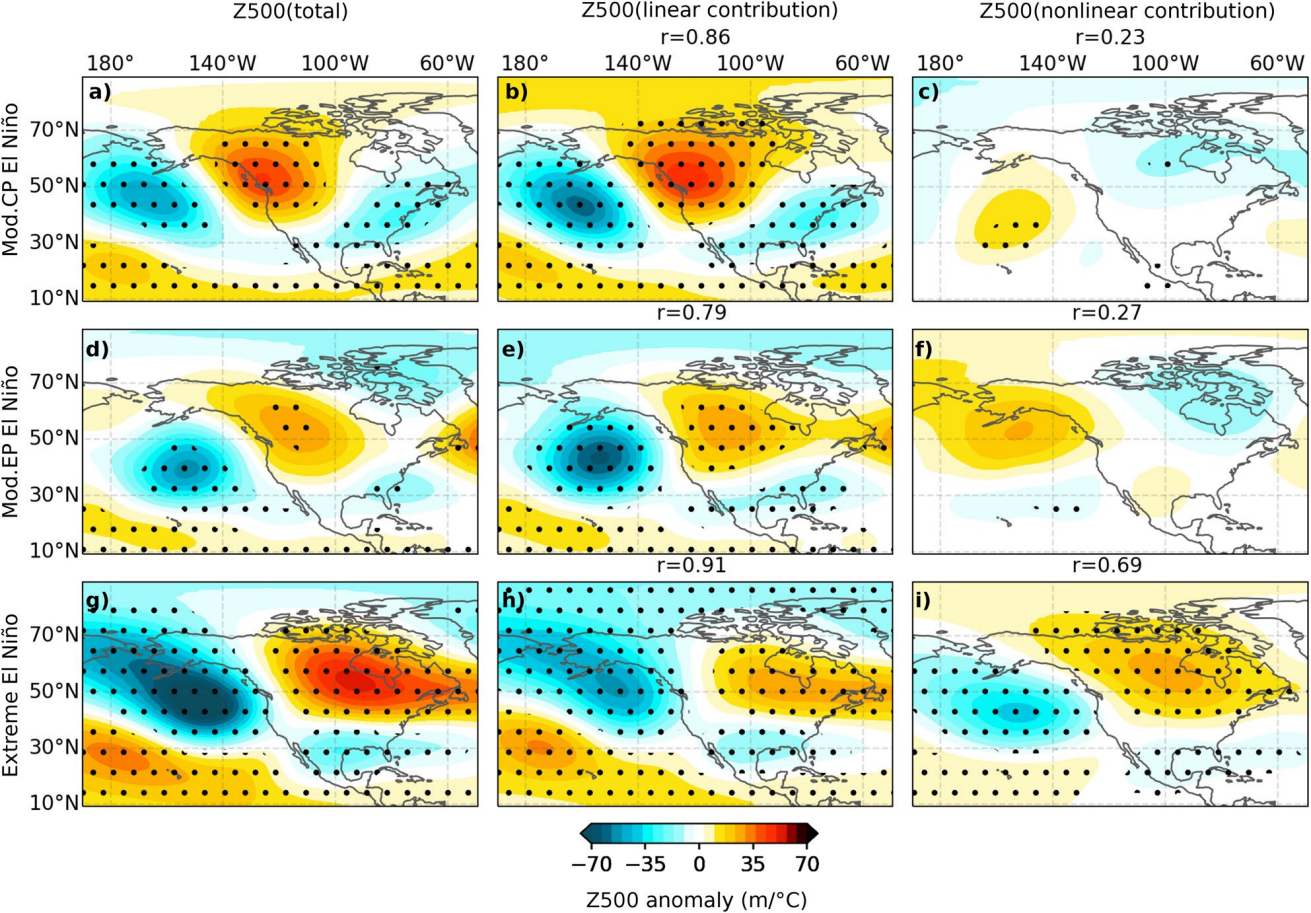
Composites of December–February (DJF) 500 hPa height anomaly from the ensemble mean CTL experiment over the 1979–2019 period for (top row) moderate Central Pacific (CP), (middle row) moderate Eastern Pacific (EP), and (bottom row) extreme El Niño events. Composites have been obtained after normalizing fields by the average [5^∘^N/S, 170^∘^E-130^∘^W] SST anomalies (considered the main forcing region, see “Methods” section), in order to highlight the pattern rather than the amplitude differences (shading, m/^∘^C). Each row consists of three panels: (left column) total anomaly, (middle column) linear response to the SST forcing and (right column) contribution from atmospheric nonlinearities. Dots indicate areas where anomalies exceed the 97.5% confidence level based on a bootstrap test with 10,000 resamples. On the middle (right) column, r indicates the pattern correlation between the total anomaly and the linear (nonlinear) SST contribution to the Z500 anomaly.

Figure 5 provides a similar analysis to Fig. 4, but for the mid-latitude response to moderate CP, moderate EP and extreme El Niño events, allowing us to explore how the aforementioned shifts in tropical precipitation influence mid-latitude teleconnection patterns. The mid-latitude response to moderate CP and EP El Niño is largely linear, resembling the PNA pattern (Fig. 5a–f). On the other hand, the extreme El Niño teleconnection pattern is primarily determined by the linear response to the eastward-shifted SST pattern (Fig. 5h vs. 5i, 0.91/0.69 pattern correlation of the linear/nonlinear contribution to total). However, as expected from the tropical rainfall response discussed earlier, the mid-latitude teleconnections associated with extreme El Niño events involve a significantly larger contribution from nonlinearities, with the intensified nonlinear rainfall in the eastern Pacific during extreme El Niño (Fig. 4i) driving an additional nonlinear response that strengthens the eastward-shifted response due to the linear contribution (Fig. 5i), almost matching the magnitude of the linear component (Fig. 5h).

In this section, we have explained why a distinct, eastward-shifted teleconnection pattern arises solely during extreme El Niño events. The SST pattern shifts eastwards during moderate EP events, but the Niño3 anomalies are still not strong enough to cross the convective threshold in the climatologically cool eastern Pacific. However, during the strongest EP events (i.e. extreme El Niño), eastward-shifted SST anomalies are strong enough to exceed the threshold for deep atmospheric convection in the Niño3 region, and atmospheric nonlinearities reinforce the eastward shift of the tropospheric heat source and of the associated mid-latitude Z500 response.

### Reproducibility of ENSO teleconnection patterns

Extratropical seasonal forecasts rely on two factors: the ability to predict ENSO and the associated tropical heat source or sink; and the reproducibility of the extratropical teleconnection pattern. The reproducibility depends on the teleconnection pattern signal to atmospheric internal variability noise ratio. In this section, we investigate the reproducibility of the teleconnection pattern we have identified for extreme El Niño against that of the PNA pattern associated with other types of ENSO events (La Niña, CP, moderate EP). As outlined in the Methods section, we have expanded the 6-member reference ensemble for the common period (1979-2014) by incorporating an additional 10 members from AMIP CMIP6 simulations, resulting in a total of 16 members for all ENSO events, except for the 2015/2016 extreme El Niño, 2016/2017 and 2017/2018 moderate La Niña events, for which only 6 members are available.

**Figure 6:**
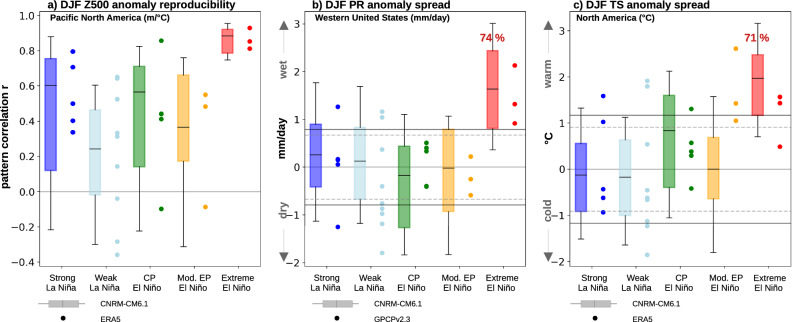
(**a**) Reproducibility of the DJF 500 hPa height pattern during ENSO events from the ensemble mean CTL experiment (whisker boxes) and ERA5 (circles), as a function of the type of ENSO event (strong La Niña, weak La Niña, CP El Niño, moderate EP El Niño, extreme EP El Niño). Whisker boxes (circles) represent the distribution of pattern correlations between individual members (individual years for ERA5) and the corresponding CTL ENSO composite, over the 20–70^∘^N, 180–60^∘^W region. Anomalies are normalized by the average [5^∘^N/S, 170^∘^E-130^∘^W] SST anomalies (considered the main forcing region, see “Methods” section), although the normalization method does not significantly affect the findings. (**b**) Distribution of the DJF rainfall anomaly average (mm/day) over the 35–50^∘^N, 130–120^∘^W region encompassing Western United States. Anomalies are not normalized. (**c**) same as (**b**) but for land surface temperature (^∘^C) over North America (40–70^∘^N, 125–80^∘^W). The whiskers, box boundaries and middle marker respectively indicate the 10th and 90th percentiles, 25th and 75th percentiles and median for the CTL ensemble, while circles indicate values for individual events in observations (ERA5 for surface temperature, GPCP v2.3 for rainfall). On panels b,c the solid/dashed grey lines indicate CTL ensemble / observed ± 0,5 * standard deviation threshold used to characterize wet/dry or warm/cool anomalies. Percentages indicate wet/dry (panel b) or warm/cold (panel c) anomalies, based on the 0.5 std threshold across all years and ensemble members (CTL ensemble). The distributions are based on 16-member ensembles except for 2015–2017 for which only 6 members are available (see “Methods” section).

We first assess the reproducibility of the Z500 patterns over the Pacific-North America region. In Fig. 6a, whiskers display the distribution of pattern correlations within 20-70^∘^N, 180-60^∘^W between each member and the composite ensemble mean for each specific ENSO phase. The circles display the same diagnostic for individual events in ERA5 correlated to the corresponding ENSO composite from the ensemble simulation. Hereafter, we refer to these quantities as the reproducibility score. First, we note that the median of the observed reproducibility reasonably matches that of the model, while its distribution falls within that of the model. This indicates that, considering the range of internal variability, the modeled teleconnection patterns satisfyingly reproduce those in observations. Secondly, the model and observed reproducibility scores exhibit a marked shift towards higher values for the extreme El Niño category (90% of modeled values, and 3 out of 3 observed values above 0.75), and a smaller spread than any other category of ENSO events. This indicates that the spatial pattern of Z500 anomalies for individual realizations is very similar to the composite extreme El Niño teleconnection in both models and observations. The Supplementary Figure S5 indicates that the extreme El Niño Z500 anomalies are both reproducible across members in the model, and across the three extreme events in both the model and observations. This enhanced reproducibility of North American Z500 anomalies during extreme El Niño events could be due to the fact that the larger amplitude, eastward-shifted response is less affected by internal atmospheric variability or other sources of external forcing, favoring a higher signal-to-noise ratio.

As mentioned in the introduction, recent studies have highlighted the association of wet conditions in California and the strongest El Niño events^34,35^. Figure 6b presents the observed and ensemble distribution of the average rainfall anomalies in the West Coast of the United States (35-50^∘^N; 130-120^∘^W, including the states of Washington, Oregon and California) for each ENSO composite phase (whisker box for the 16 model realizations distribution, and circles for observed events). Despite the limited number of realizations (3 to 9 depending on the ENSO type), the observed distributions are generally reasonably consistent with the observed ones. Extreme El Niño years display the most pronounced shift in the rainfall probability distribution over the Western United States, with a wet anomaly (defined based on 0.5 standard deviation threshold) in 74% (see Fig. 6b) of the 38 extreme El Niño ensemble members. The 3 observed extreme El Niño events also correspond to wet anomalies, further underlining the consistent impact of extreme El Niño on Californian rainfall in observations. Figure 6c presents the same diagnostic for Ts anomalies over North America (within 40–70^∘^N, 125–80^∘^W) and leads to similar conclusions. The North American warming during extreme El Niño is robust, with 71% of the members exhibiting warm anomalies that exceed 0.5 standard deviation in our simulation, and a warm anomaly for 2 of the 3 observed events. One can however note that the 3 moderate EP events also correspond to warm anomalies in observations, unlike in the model.

In this section, we have shown that the boreal winter Z500 anomalies are more alike the composite teleconnection patterns for extreme El Niño than for any other type of ENSO event (La Niña, CP, moderate EP). In particular, this specific teleconnection pattern leads to more consistent wet anomalies over Western United States and warm anomalies over North America. The implications of these findings for seasonal forecasts will be discussed below.

## Discussion

In this study, we use observations and AMIP-type ensemble integrations with the CNRM-CM6.1 ARPEGE-Climat v6.3 atmospheric general circulation model to explore the diversity of DJF ENSO teleconnection patterns over the North Pacific and North America. Previous studies have explored differences in teleconnection patterns between El Niño and La Niña^12^, between central and eastern Pacific El Niño events^27–29^, or between El Niño events with and without convective anomalies in the eastern-central Pacific^36–38^. This last category is closest to our extreme El Niño definition, but also retains moderate EP events that do not involve as large eastern Pacific rainfall anomalies as extreme El Niño events do. We show that the differences in teleconnection pattern discussed in previous studies are in fact solely due to extreme El Niño events (see Supplementary Figure S3). These events display a unique eastward shift relative to the well-known PNA pattern, resulting in a pattern that closely resembles the TNH teleconnection pattern^43,44^. On the other hand, asymmetries between the La Niña and moderate El Niño teleconnection patterns are not statistically significant. Similarly, differences between the moderate CP and moderate EP teleconnection patterns are much less significant than those between extreme El Niño events and any other category. Our results however suggests statistically significant differences between the moderate CP and moderate EP teleconnection patterns over the North Pacific and western North Atlantic regions. This would need to be investigated in a larger ensemble, as we only have three moderate EP events in our sample, and a large sampling size is needed to reach statistical robustness^15,45^. Investigating moderate CP/EP teleconnection diversity in large AMIP ensembles would be a pertinent perspective for accurately evaluate changes in teleconnections between these two specific phases.

Our record being quite short (only 3 extreme El Niño events), it is difficult to definitively conclude that the north American ENSO teleconnection distribution is bimodal, with the eastward shift only occurring during extreme El Niño events. There may be a more continuous eastward shift of the teleconnection pattern as SST and precipitation anomalies in the east progressively increase, aligning with the ideas of some previous publications^36–38^. However, because we cannot detect it based on last 40 years record, the idea that the ENSO teleconnection pattern only changes for extreme El Niño events can serve as a good null hypothesis.

Several studies^12,17^ have emphasized the role of atmospheric nonlinearities related to the threshold for atmospheric convection, as opposed to the influence of the different SST patterns during La Niña, CP or EP events. Our methodology allows us to disentangle the respective contributions of these two factors. As a previous study^32^, we find that the SST pattern and atmospheric nonlinearities both influence changes in ENSO teleconnection patterns. For moderate EP El Niño events, the eastward-shifted SST anomalies do not exceed the convective threshold over the Niño3 region. Conversely, during extreme El Niño events, the SST anomaly is both eastward-shifted and sufficiently large to cross the convective threshold over much of the Niño3 region. Consequently, atmospheric nonlinearities combine with the linear response to SST to produce a tropospheric heat source shift from the central to the eastern Pacific, leading to an eastward shift in the North American teleconnection pattern.

Our study contributed to better understand the mechanisms behind the tropospheric heat source change, but did not investigate the Rossby wave pathway between this eastward-shifted heat source and the mid-latitudes. A previous study using an idealised atmospheric model^46^ found a sensitivity of the mid-latitude teleconnection pattern to the longitude of the equatorial heat source that is reminiscent of that we highlighted during extreme El Niño events. Following this perspective, sensitivity experiments with atmospheric GCMs forced with various SST patterns such as the Green’s Function Model Intercomparison Project (GFMIP^47^) or Rossby way tracing analysis could provide valuable insights into the mechanisms involved in the teleconnection change during extreme El Niño events.

Finally, we examined the reproducibility of North American 500 hPa geopotential height anomalies for various ENSO event categories, and found they were more reproducible during extreme El events than during any other type (La Niña, CP, moderate EP). This is attributable to a more favorable signal-to-noise ratio due to the larger amplitude forcing in the tropics, although this hypothesis will have to be investigated. This finding carries important implications for seasonal climate predictability over North America. The consistently higher risks of wet conditions over Western United States and warm conditions over Canada and Northern US during extreme El Niño are potentially predictable, provided that an extreme El Niño can be skillfully forecasted. A recent study indicates that the amplitude of extreme El Niño events is strongly affected by the semi-random occurrence of Westerly Wind Events during summer and fall in the equatorial Pacific^48^. An investigation of the lead-time at which extreme El Niño conditions can confidently be predicted is thus necessary to evaluate the potential skill gain from considering the unique extreme El Niño teleconnection pattern.

Our results also have implications for the future evolution of ENSO impacts. Recent studies suggested an eastward shift of the ENSO-related tropical rainfall and extratropical teleconnections^49^, as well as more frequent extreme El Niño events^50^ under warmer climates. Our results imply that this should be associated with more frequent wet conditions over Western United States including California, and heat waves over Northern US and Canada. In the future, we will explore whether CMIP6 models reproduce the specific extreme El Niño teleconnection pattern discussed in the present study, and the consequences for the future projections of ENSO events impacts over North America.

## Methods

We use AMIP ensembles designed by Srinivas et al, (in revision)^42^ with the atmospheric component of the Centre National de Recherches Météorologiques (CNRM) - CERFACS climate model CNRM-CM6.1, ARPEGE-Climat v6.3^51^. It is a global spectral model with a T127 triangular truncation which is equivalent to a spatial resolution of about 150 km. The vertical coordinate is progressive hybrid sigma-pressure, from 0.01 hPa to the surface over 91 levels. This model is able to reproduce the ENSO observed sea level pressure and air temperature teleconnections to the North Pacific^52^.

Our control simulation (abbreviated as CTL) is the AMIP-type 6-member ensemble experiment run over 1979-2019 by Srinivas et al. (in revision)^42^. Initial conditions are selected as in Voldoire et al. (2019)^52^, following Rhoerig et al. (2020)^51^. Figures 1, 2, 3, 4 and 5 use 6-members ensemble. This ensemble was too small to assess the reproducibility of the teleconnection patterns (Fig. 6) and we increased the size of the ensemble by using the 1979-2014 10-member AMIP experiment of Voldoire et al. (2019)^52^. Figure 6 hence uses 16 members for all events except for 2015-2016, for which only 10 members are available. Figure S6 illustrates that the Figs. 1, 2, 3 are robust when using 16 instead of 6 members. We did however stick to 6 members in the main text so that the linear/nonlinear decomposition of Figs. 4 and 5 is comparable to Figs. 1, 2 and 3.

For 500 hPa geopotential height and surface temperature, we used the monthly averaged ERA5 on pressure levels^53^ as a reference dataset. Our monthly precipitation reference dataset is the Global Precipitation Climatology Project v2.3^54^. All model and observed anomalies are obtained after linearly detrending and removing the 1979-2019 climatology. In this manuscript, we only discuss boreal winter (December-January-February, DJF) average anomalies, as ENSO SST anomalies peak in boreal winter^55^ and the PNA teleconnection pattern in February^8^.

We estimate the linear and nonlinear components of the atmospheric response to SST anomalies following the methodology of Srinivas et al (in revision^42^). The CTL ensemble mean is our estimate of the forced response to the SST anomaly $T^\prime$ , noted $M(T^\prime )$. Observed interannual SST anomalies relative to the 1979-2019 climatology are subtracted from the climatology, and used to force a second 6-member ensemble experiment over 1979-2019, and the resulting ensemble-mean forced response is noted $M(-T^\prime )$. By calculating the half-difference $\frac{1}{2}(M(T^\prime )-M(-T^\prime ))$, Srinivas et al (in revision)^42^ effectively isolates the odd terms of the Taylor series (i.e. linear term, plus all non-symmetry-breaking nonlinearities). Conversely, the half-sum $\frac{1}{2}(M(T^\prime )+M(-T^\prime ))$ extracts all the even terms (symmetry-breaking nonlinearities). As demonstrated by Srinivas et al (in revision)^42^, $\frac{1}{2}(M(T^\prime )-M(-T^\prime ))$ is dominated by the first order linear term, and $\frac{1}{2}(M(T^\prime )+M(-T^\prime ))$ by the second-order quadratic term. In the paper, we simply refer to these as the linear and nonlinear contributions, respectively.

ENSO events are defined according to the NOAA oceanic Niño index (ONI^56^), when the 3-month running mean of mean ERSST.v5 SST anomalies over the Niño-3.4 region (5^∘^N-5^∘^S, 120^∘^-170^∘^W) exceeds 0.5^∘^C (El Niño) or falls below -0.5^∘^C (La Niña). To compute the SST anomalies for a consecutive 5-year periods, a 30-year base periods is employed as climatology^57^. The CP/EP classification follows the “Cold Tongue/Warm Pool” classification method^33^, based on coordinate transformation of the Niño3/Niño4 phase space to calculate the CT/WP indices. Extreme El Niño events are defined as events with DJF ONI above +2^∘^C, according to recent studies^58,59^. Strong La Niña events are defined as events with DJF ONI below -1.5^∘^C, according to the Climate.gov ENSO Blog^60^. Supplementary Figure S4 lists the events in each category, and summarizes how some categories are subset of some others (e.g. extreme El Niño are included in EP).

We use composites to isolate the tropical rainfall response and extratropical teleconnections associated with various event categories (La Niña, El Niño, CP, moderate EP, extreme El Niño). We identify composite anomalies that are different from zero at a 97.5% confidence level based on a bootstrap method. Multiple composites are built by randomly resampling (10, 000 times for the model, 1000 for observations) the sample (one sample per event for observations, as many samples as ensemble members per type for the model). The lower (upper) 2.5% of the distribution allow to determine whether the mean is significantly above (below) from 0.

Figures 3, 4 and 5 display differences between composites. They aim at identifying differences in spatial pattern rather than differences in amplitude of the atmospheric response. The events have thus been normalized by the amplitude of the SST forcing prior to computing the composites and their difference. We have chosen to normalize based on average SST anomalies over the [5^∘^N/S, 170^∘^E-130^∘^W] region, that covers most of the more conventional Niño3.4 and Niño4 boxes (except the most eastward and westward 10^∘^). We chose this region because it encompasses both the regions of maximum La Niña and El Niño precipitation anomalies, and hence broadly the region where SST anomalies force the atmosphere. Note, however, that the results are not sensitive to using, e.g., the more conventional Niño3.4 box for normalizing.

### Supplementary Information

Supplementary Information.

## Data Availability

Figures are prepared in Python, codes are available on request from MB. The simulations are available on request from GS.
