# Supplementary material for: A distinct and reproducible teleconnection pattern over North America during extreme El Niño events
Source: Sci Rep. 2024 Jan 30;14:2457. doi: 10.1038/s41598-024-52580-9 (PMC10828491; doi:10.1038/s41598-024-52580-9)
Supplement: Supplementary file 1 — Supplementary Information. [file 41598_2024_52580_MOESM1_ESM.pdf]

Supplementary Information for:

A distinct and reproducible teleconnection pattern over North America during extreme El Niño events

Margot Beniche[1]*, Jérôme Vialard[2], Matthieu Lengaigne[3], Aurore Voldoire[4], Gangiredla Srinivas[5], Nicholas M.J. Hall[1]

[1] LEGOS, CNRS/CNES/IRD/Université de Toulouse, Toulouse, France
[2] LOCEAN-IPSL, CNRS/IRD/MNHN/Sorbonne Université, Paris, France
[3] MARBEC, CNRS/IFREMER/IRD/Université de Montpellier, Sète, France
[4] CNRM, Météo-France/CNRS/Univeristé de Toulouse, Toulouse, France
[5] CSIR-National Institute of Oceanography, Dona Paula, Goa, India

Submitted to Scientific Reports
22 September 2023

* Corresponding author :
M. Beniche
PhD student - LEGOS
margot.beniche@univ-tlse3.fr

This supplementary material contains six figures (S1 to S6) and their caption. Below each figure, a short text in italics summarizes the main take-home message from this figure and how it relates to the figures in the article.

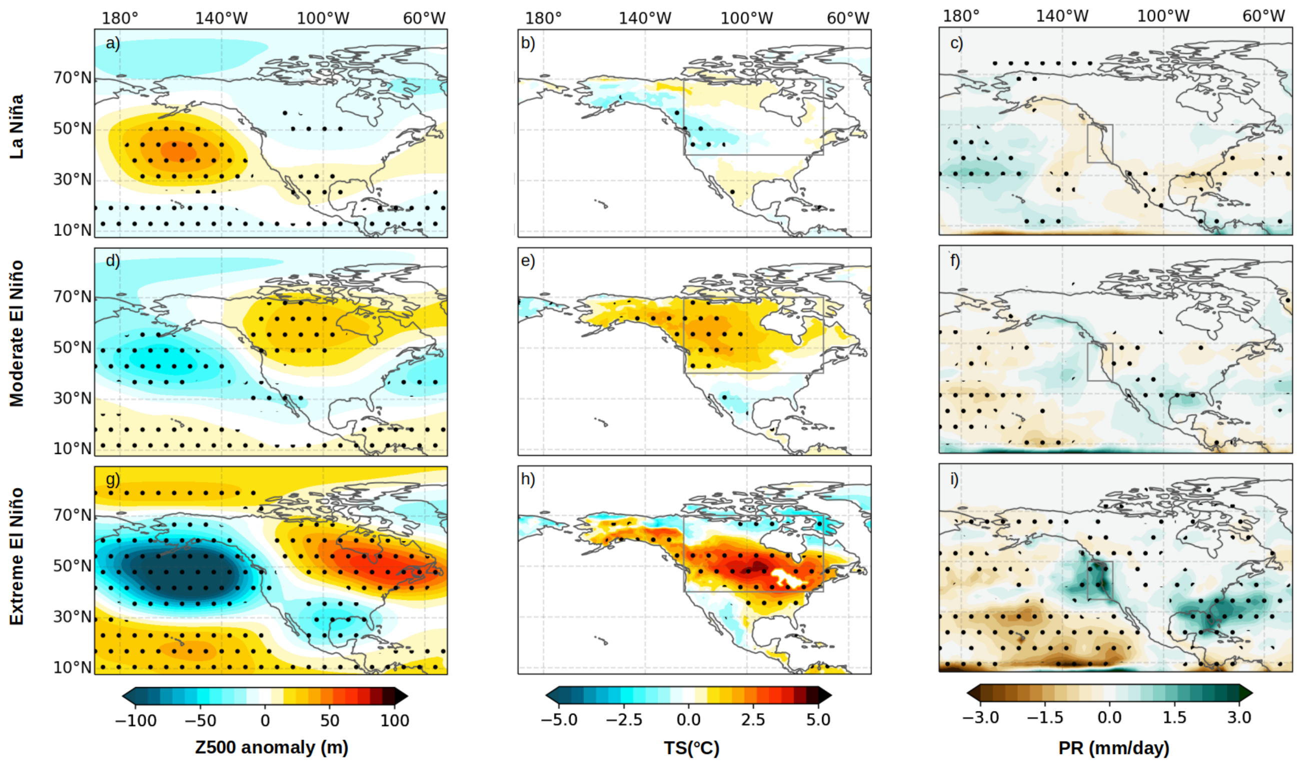

Supplementary Figure S1 : Composites of (left column) 500 hPa height (shaded, m), (middle column) land surface temperature (shaded, °C) and (right column) rainfall (shaded, mm/day) December-February (DJF) anomalies from ERA5 (left and center) and GPCP V2.3 (right) over the 1979-2019 period for (top row) La Niña, (middle row) moderate El Niño and (bottom row) Extreme El Niño events. The composites are not normalized. Dots indicate regions where anomalies exceed the 97.5% confidence level based on a bootstrap test with 1,000 resamples. Boxes in the middle column indicate the North-east America box (30-60°N, 100-60°W) used in Fig6c. Boxes in the right column indicate the Western United State box (35-50°N, 130-120°W) in Fig6b.

To compare with model (Fig2). We can note the good performance of the model to reproduce observed teleconnection for different variables. It should however be noted that the observed composites are less statistically significant, since they are not based on an ensemble average (16 members over 1979-2015 and 6 over 2015-2019) as for the model.

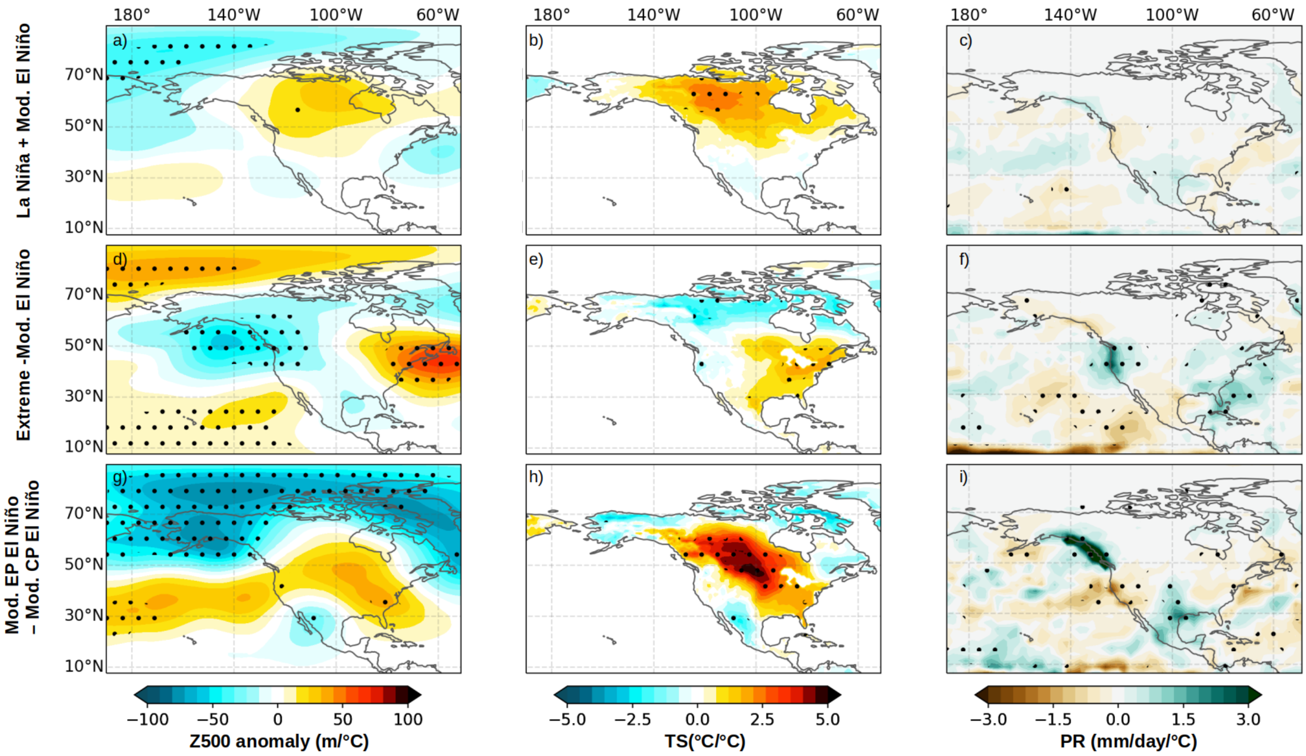

Supplementary Figure S2 : Composites sum/differences of (left column) 500 hPa height (shaded, m/°C), (middle column) land surface temperature (shaded, °C/°C) and (right column) rainfall (shaded, mm/day/°C) December-February (DJF) anomalies from ERA5 (left and center) and GPCP V2.3 (right) over the 1979-2019 period for (top row) La Niña plus El Niño, (middle row) Extreme minus Moderate El Niño and (bottom) Moderate Eastern Pacific minus Central Pacific. Each composite is obtained after normalizing by SST anomalies in the forcing region ([5°N/S, 170°E-130°W], see Methods) in order to emphasize pattern rather than amplitude differences. Dots indicate areas where anomalies exceed the 97.5% confidence level based on a bootstrap test with 1,000 resamples.

To compare with model (Fig3). Unlike the model, observations present noiser composites due to extratropical internal variability and the difference between composites that are displayed here are less statistically significant. The significant difference anomalies plotted in the Mod. EP El Niño minus Mod. CP El Niño are found to not be linked to ENSO-related activity, and year by year observed composites seems to display a strong NAO-related activity over North Atlantic, absent in the model. Despite those differences, there is generally a qualitative agreement between the model and observed composites.

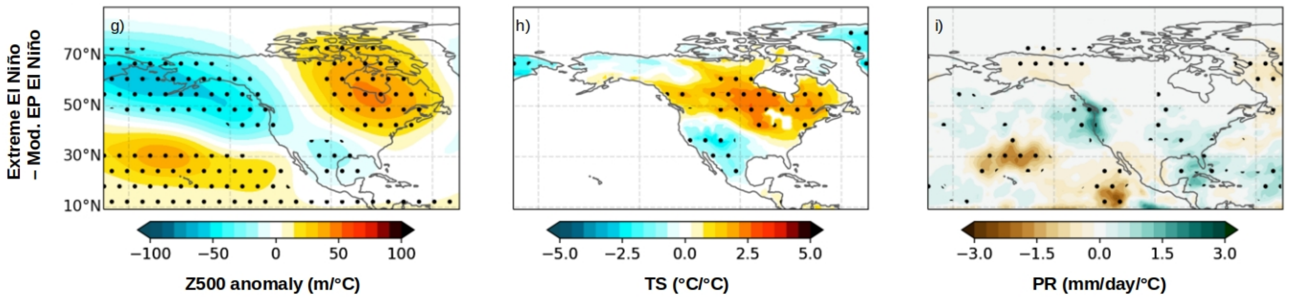

Supplementary Figure S3 : Composites sum/differences of (left column) 500 hPa height (shaded, m/°C), (middle column) land surface temperature (shaded, °C/°C) and (right column) rainfall (shaded, mm/day/°C) December-February (DJF) anomalies from the ensemble mean CTL experiment over the 1979-2019 period for Extreme minus Moderate Eastern Pacific El Niño. Each composite is obtained after normalizing by SST anomalies in the forcing region ([5°N/S, 170°E-130°W], see Methods) in order to emphasize pattern rather than amplitude differences. Dots indicate areas where anomalies exceed the 97.5% confidence level based on a bootstrap test with 10,000 resamples.

This figure complements Fig3, by showing Extreme EP minus Moderate EP composites (Figure 3 displays Extreme EP minus all moderate El Niño events). It demonstrates that the eastward shift of the SST pattern alone during moderate Eastern Pacific events is not sufficient to trigger distinct teleconnection patterns.

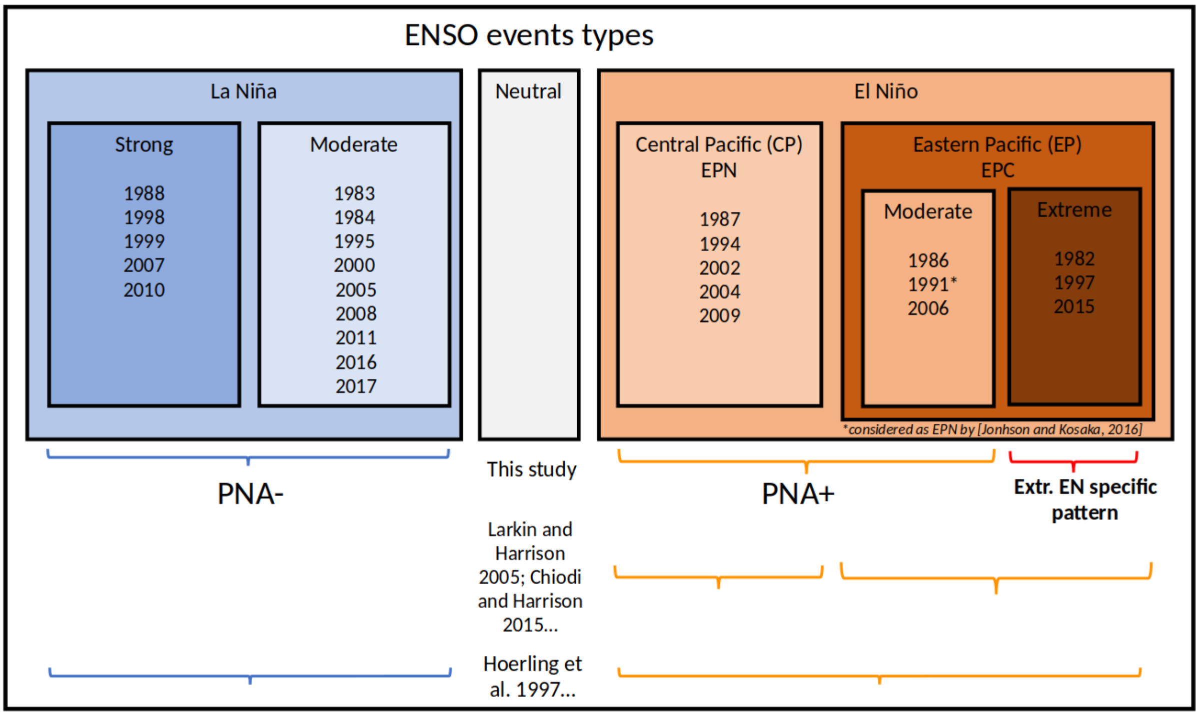

Supplementary Figure S4: List of ENSO events categories over the 1979-2019 period used in this paper (the year indicate the onset year, e.g. 1997 for 1997-98). See the methods section for the references of the method used to obtain each category: La Niña (strong and moderate La Niña categories are only distinguished on Fig 6), Neutral, and El Niño including: Central Pacific (CP) and Eastern Pacific (EP). The CP and EP categories respectively match the non-convective (EPN) and convective (EPC) categories used in [Jonhson and Kosaka, 2016], except for 1991 (considered as non-convective by [Jonhson and Kosaka, 2016] and EP here). We also distinguish extreme El Niño events, which are a subset of the EP events for the period we consider.

The bottom part of the sketch summarizes how differences in teleconnection patterns previously attributed to the ENSO phase (El Niño vs La Niña, e.g. Hoerling et al. 1997) or ENSO diversity (CP vs EP, e.g. Larkin and Harrison 2005, etc…) are in fact largely attributable to the specific extreme El Niño teleconnection pattern. Potential differences between EP and CP teleconnection patterns appear to be much weaker and not detectable from observations or our ensemble simulation dataset.

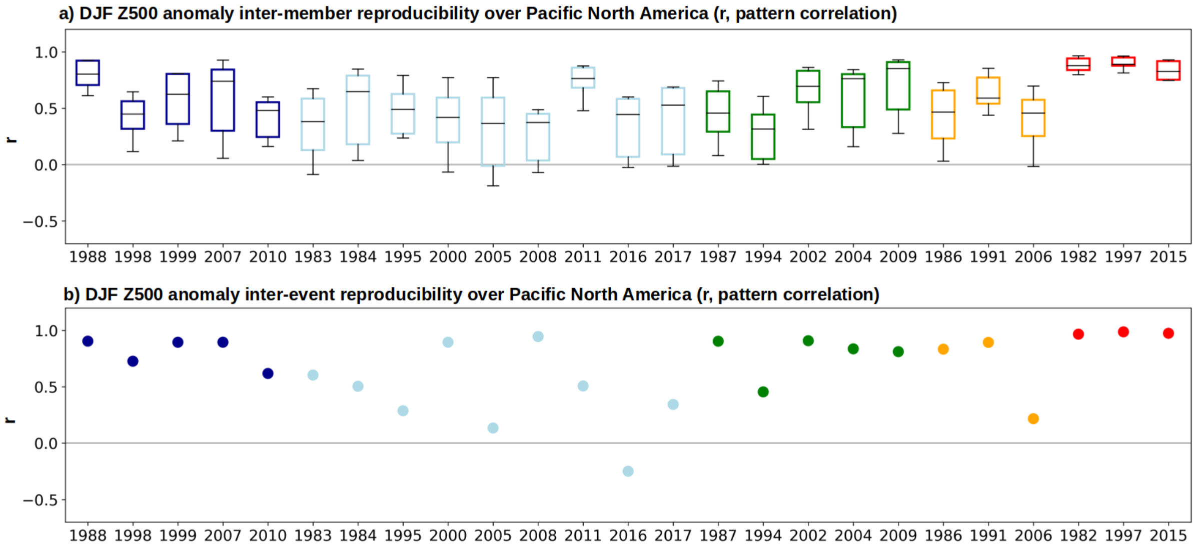

Supplementary Figure S5 : (a) Inter-member reproducibility of the DJF 500 hPa height pattern amongst ensemble members. Whisker boxes represent the distribution of pattern correlations between individual members and the corresponding ensemble mean for that year, over the 20-70°N, 180-60°W region. (b) Inter-event DJF 500 hPa pattern reproducibility. The circle represents the pattern correlation (20-70°N, 180-60°W region) between each event ensemble mean and the corresponding CTL ENSO composite. All values are normalized by the mean SST anomaly in region [5°N/S, 170°E-130°W], although the normalization method does not significantly affect the findings. The whiskers, box boundaries and middle marker respectively indicate the 10th and 90th percentiles, 25th and 75th percentiles and median. The distributions are based on 16-member ensembles except for 2015-2019 for which only 6 members are available (see Methods).

This figure complements figure 6a. Figure 6a indicates that the 500 hPa height anomalies over North America in any member is very similar to the extreme El Niño composite teleconnection pattern. The figure S4 illustrates that this strong reproducibility both results from a strong inter-member reproducibility during a given extreme El Niño (panel a) and from the similarity of the North American anomaly pattern during the three extreme El Niño which where considered (panel b).

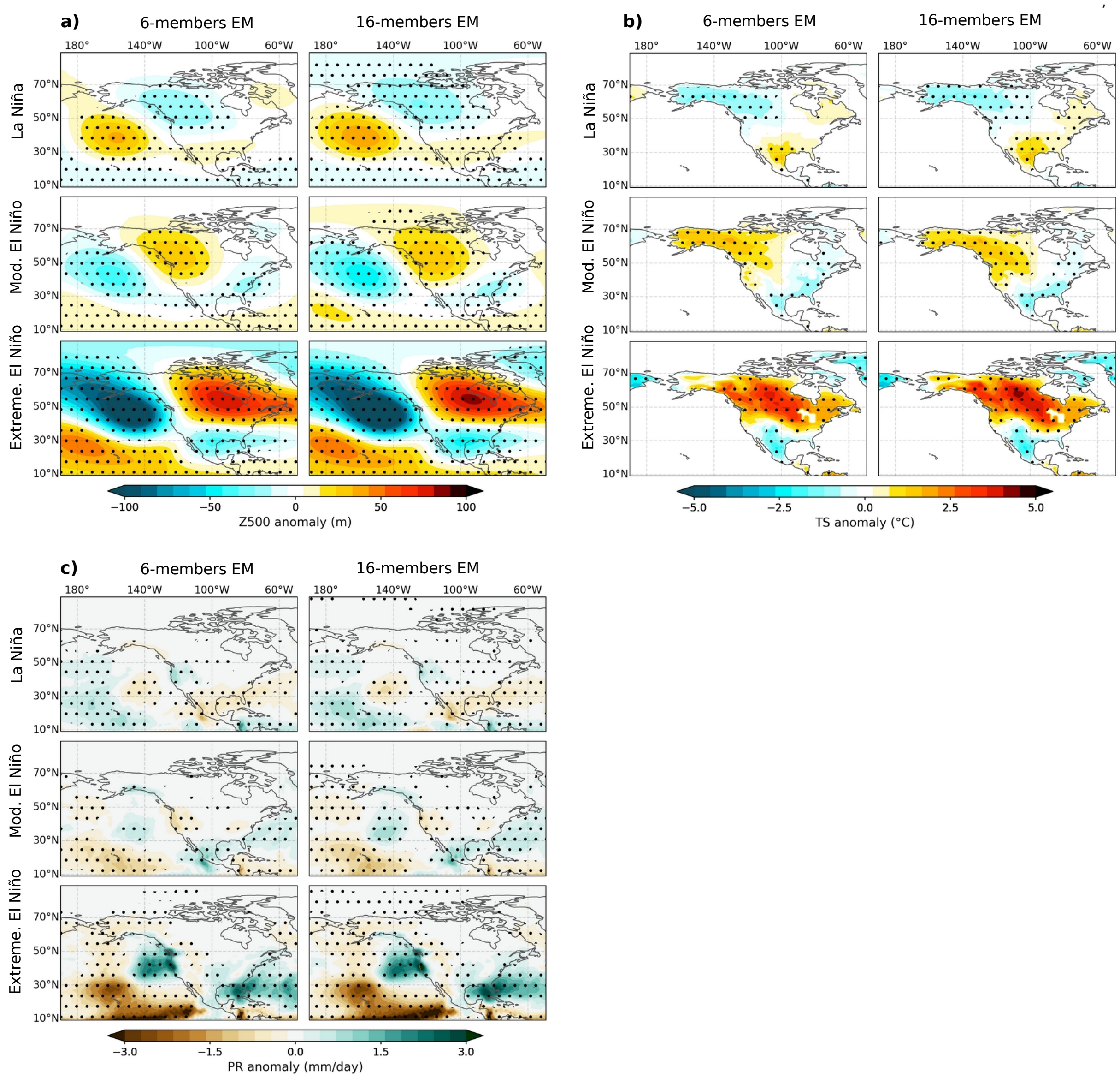

Supplementary Figure S6 : Composites of (a) 500 hPa height (shaded, m), (b) land surface temperature (shaded, °C) and (c) rainfall (shaded, mm/day) December-February (DJF) anomalies from the ensemble-means 6-member ensemble CTL experiment (right) and extended 16-member ensemble experiment (left) over the 1979-2019 period for (top row) La Niña, (middle row) moderate El Niño and (bottom row) Extreme El Niño events. The composites are not normalized. Dots indicate regions where anomalies exceed the 97.5% confidence level based on a bootstrap test with 10,000 resamples.

This figure demonstrates that the composites of Figure 2 of the main text, obtained with 6 members, are robust when recomputed using 16 members (see Methods).
